# A single-nucleotide polymorphism rs708567 in the IL-17RC gene is associated with a susceptibility to and the curve severity of adolescent idiopathic scoliosis in a Chinese Han population: a case-control study

**DOI:** 10.1186/1471-2474-13-181

**Published:** 2012-09-21

**Authors:** Song Zhou, Xu-sheng Qiu, Ze-zhang Zhu, Wei-fei Wu, Zhen Liu, Yong Qiu

**Affiliations:** 1 Spine Surgery Department, the Affiliated Drum Tower Hospital of Nanjing University Medical School, Nanjing, China

**Keywords:** Adolescent idiopathic scoliosis, IL-17RC, Single-nucleotide polymorphism

## Abstract

**Background:**

Although the pathogenesis of adolescent idiopathic scoliosis (AIS) remains controversial, genetic factors are thought to play key roles in the development of AIS. In a recent genome-wide association study, a polymorphism in the interleukin-17 receptor C (IL-17RC) gene was reported to be associated with the susceptibility to AIS, implicating IL-17RC as a novel predisposing gene for AIS. However, as this association has not been replicated in other populations, its global applicability remains unclear.

**Methods:**

A total of 529 Chinese girls with AIS and 512 healthy age-matched controls were recruited in this case–control study from June 2007 to December 2009. Polymerase chain reaction restriction fragment length polymorphism (PCR-RFLP) analysis was performed to detect the genotype of the single-nucleotide polymorphism (SNP) rs708567 in the IL-17RC gene. Case–control and case-only studies were performed to determine the association between the IL-17RC gene polymorphism and the susceptibility to and curve severity of AIS.

**Results:**

The GG genotype and G allele frequencies were significantly higher in the AIS patients than in the controls (*χ*^2^ test: P = 0.023 and 0.028, respectively). The risk for the GG genotype is 1.550-fold (95% CI: 1.062 - 2.261) higher than the AG genotype, and the risk for the G allele is 1.507-fold (95% CI: 1.046 - 2.172) higher than the A allele. Additionally, a subgroup of skeletally mature AIS patients (n = 241) who carried the GG genotype showed a significantly higher mean maximum Cobb angle than those carrying the AG genotype (36.01 ± 13.12° vs. 28.92 ± 7.43°, P = 0.007).

**Conclusions:**

This study confirms the significant association between the IL-17RC gene polymorphism and the susceptibility to and curve severity of AIS in a Chinese Han population, suggesting that the IL-17RC gene is an AIS-predisposing gene in Chinese Han population.

## Background

Adolescent idiopathic scoliosis (AIS) is one of the most common spinal deformities, with a world-wide pediatric prevalence of 2-3% [1]. In recent decades, many etiological factors have been proposed for AIS, such as uncoupled growth of the spinal column [2], paraspinal muscle dysfunction[3,4], hormonal disturbances [5-7], and inflammation[8]. However, no single factor can explain all of the clinical characteristics of AIS. Therefore, AIS is generally regarded as a multi-factorial disorder for which hereditary or genetic factors likely play important roles.

The genetic factors contributing to AIS have been described in many studies. Genome-wide linkage analysis studies have identified several variations in chromosomal regions, including 9q31.2–q34.2, 12p, 17p11 and so on [9-14], as being associated with an increased susceptibility to AIS. Through genetic association studies, several genes, such as tryptophan hydroxylase 1 (TPH1) [15], estrogen receptor α (ESR1) [16], and the matrilin-1 gene (MATN1) [17], have been identified as genes that predispose an individual to AIS. Furthermore, a number of genes have also been found to be related to the curve severity of AIS[18,19].

In a recent genome-wide association study using data from 137 AIS patients of European ancestry, Dormans JP et al.[20] reported that SNP rs708567 (a common missense mutation) was associated with susceptibility to AIS. SNP rs708567 is located on chromosome 3p25.3 and occurs in an exon of the IL-17 receptor C (IL-17RC) gene, which codes for an integral member of the IL-17R complex [21]. As an essential part of the IL-17 cytokine/IL-17R signaling axis, the IL-17R complex mediates the signal transduction of the IL-17 cytokine family, which promotes the production of pro-inflammatory cytokines, such as tumor necrosis factor α (TNF-α), interleukin-1β (IL-1β), and interleukin-6 (IL-6) [21]. The findings of Dormans JP et al. [20] identified IL-17RC as a novel predisposing gene for AIS, thus providing significant information regarding the genetic etiology of AIS.

However, due to the small sample sizes, population stratification, variability in the control population, and racial and ethnic differences, genetic association studies may produce spurious associations [22]. Therefore, the purpose of this case–control study was to replicate the previously reported association between the IL-17RC gene polymorphism and the susceptibility to AIS and to further investigate the contribution of this polymorphism to the curve severity of AIS within a Chinese Han population. Our results suggest that the IL-17RC gene is an AIS-predisposing gene in Chinese Han population.

## Methods

This study included two sections: a case–control study and a case-only study.

The case–control study evaluated the genetic association between the predisposition to AIS and the rs708567 SNP within the IL-17RC gene. The case-only study evaluated the genetic association between rs708567 and curve severity in a subgroup of AIS patients who had reached skeletal maturity. Skeletal maturity was defined as being at least 16 years old and having a Risser sign of 5 (the Risser sign is a grading system for the ossification of the iliac apophysis)[23]. As treatments may change the natural history of AIS, patients who had received any previous treatment were initially excluded from the case-only study.

### Subjects

A total of 698 Chinese girls with AIS, aged 11 to 18 years, and 512 gender- and age-matched controls were consecutively recruited at our scoliosis clinic from June 2007 to December 2009. A diagnosis of AIS was made according to a clinical examination and standard standing posteroanterior whole-spine radiography. The scoliosis curve severity was measured according to the Cobb method, and patients (529 cases) with a major Cobb angle ranging from 20° to 100° were included in the study. Patients (104 cases) with a curve less than 20° were excluded because of a possible “false-positive” diagnosis of AIS, and patients (65 cases) with a curve greater than 100° were also excluded because of a possible association with intraspinal and brain stem abnormalities. In addition, patients who were diagnosed with congenital scoliosis, neuromuscular diseases, endocrine diseases, skeletal dysplasia, connective tissue abnormalities or scoliosis secondary to some other disorder were excluded from the subset. The healthy controls were recruited during a regular school health-screening program. To rule out any spinal deformity, the Adam forward-bending test [24] was performed by an experienced orthopedic surgeon for all of the controls. In addition, no subjects showed evidence of bone disease or growth disturbances, systemic illness or other conditions known to affect bone metabolism or had a history of recent steroid intake.

The study protocols were approved by the University and Hospital Ethics Committee. Informed consent was obtained from all of the subjects and their parents prior to the DNA analysis.

### Blood sampling and genotyping

As described by Dormans JP et al. [20], SNP rs708567 in the IL-17RC gene is associated with a susceptibility to AIS. The present study focused only on this SNP, and rs708567 genotyping was performed using PCR-RFLP (polymerase chain reaction restriction fragment length polymorphism) in the Chinese Han population described above.

The total genomic DNA was extracted from 5 to 7 mL of ethylenediaminetetraacetic acid anti-coagulated whole blood from each patient using genomic DNA isolation kits (Amersham, Buckinghamshire, UK) according to the manufacturer’s instructions. The DNA samples were frozen and stored until needed. Genotyping was performed using PCR-RFLP. The primers are listed in Table 1. The genomic DNA was amplified in a PCR volume of 10 μL that contained 5 μL 2 × BenchTopTM Taq Master Mix, 0.2 μL each primer (1.0 μmol/L), 3.6 μL ddH_2_O and 1.0 μL genomic DNA (1 μg). A typical PCR amplification program consisted of 40 amplification cycles of 30 s at 96°C, 45 s at the primer-appropriate annealing temperature, and 30 s at 72°C. A final elongation step was performed for 7 min at 72°C. For restriction enzyme digestion, the PCR product was incubated with 4 U of HinfI for 4 h in the presence of the accompanying buffer. The digested PCR products were visualized by electrophoresis using a 3.5% agarose gel. The genotyping results were validated by duplicate genotyping with 10% of the samples (Table 1).

**Table 1:** Primers and restriction enzymes for PCR-RFLP analysis

**SNP**	**Primers**	**Annealing Temperature (°C)**	**PCR Product Size (bp)**	**Restriction Enzyme**	**Fragment Size After Enzymatic Cleavage (bp)**
rs708567	Forward Primer: 5’-AGTAGGGTAGGCCTGGAAGG-3’	57	210	HinfI	GG: 210
	Reverse Primer: 5’-CACTGGGAAGAGCCTGAAGA-3’				AG: 161 + 49

*PCR-RFLP, polymerase chain reaction restriction fragment length polymorphism; SNP, single-nucleotide polymorphism.

### Statistical analyses

A power analysis was performed using PASS (version 11; NCSS, Kaysville, UT; http://www.ncss.com/pass.html). The other statistical analyses were performed using SPSS 13.0 for Windows (SPSS Inc., Chicago, IL). Hardy-Weinberg equilibrium (HWE) was tested by a goodness-of-fit *χ*^2^ test. The case–control difference for the allele and genotype frequencies was analyzed using the *χ*^2^ test. The odds ratios (ORs) and 95% confidence interval (95% CI) ranges were calculated. A one-way analysis of variance was used to compare the mean maximum Cobb angle of the different genotypes in the AIS patients. A P-value lower than 0.05 was considered to be statistically significant.

## Results

A total of 529 Chinese AIS girls with a mean age of 14.54 ± 1.62 years and 512 gender- and age-matched controls with a mean age of 14.36 ± 1.93 years were recruited for this study. The mean Risser sign of the AIS patients and the mean maximum Cobb angle were 3.17 ± 1.89 (range 0–5) and 38.30 ± 16.71° (range 20° - 100°), respectively. Our sample size had an 81% power to detect the AIS-relevant SNP within the IL-17RC gene. The genotype frequencies showed no significant deviation from Hardy-Weinberg equilibrium (HWE) in either the AIS group or control group. Of these 529 AIS patients, 241 patients (mean maximum Cobb angle of 35.24 ± 12.81°) who had reached skeletal maturity were included in the case-only study.

### Association of SNP rs708567 with the susceptibility to AIS

The genotype and allele frequencies of SNP rs708567 in the AIS patients and controls are shown in Table 2. Overall, the frequencies of the GG genotype and the G allele in the AIS patients were significantly higher than in the controls (GG genotype, 90.17% vs. 85.55%, respectively, P = 0.023; G allele, 95.1% vs. 92.8%, respectively, P = 0.028). The risk for the GG genotype is 1.550-fold higher (95% CI: 1.062 - 2.261) than the AG genotype, and the risk for the G allele is 1.507-fold higher (95% CI: 1.046 - 2.172) than the A allele.

**Table 2:** Distribution of the genotype and allele frequencies of SNP rs708567 in the IL-17RC gene in AIS patients (n = 529) and controls (n = 512)

**SNP**	**Genotype/Allele**	**Group, n (%)**		**P-value**	**OR (95% Confidence Interval)**
		**AIS**	**Controls**		
rs708567	GG	477 (90.17%)	438 (85.55%)	0.023	1.550 (1.062 - 2.261)
	AG	52 (9.83%)	74 (14.45%)		0.645 (0.442 - 0.941)
	AA	-	-		-
	G	1006 (95.1%)	950 (92.8%)	0.028	1.507 (1.046 - 2.172)
	A	52 (4.9%)	74 (7.2%)		0.664 (0.460 - 0.956)

OR, odds ratio. The data are presented as the no. of individuals and their percentage of the population tested. A value of P < 0.05 (*χ*^2^ test) was considered to be statistically significant.

### Association of SNP rs708567 with curve severity in AIS

We evaluated the association of SNP rs708567 with curve severity in a subset of AIS patients who had reached skeletal maturity (n = 241) in a case-only study. Overall, AIS patients with the GG genotype showed a significantly higher mean maximum Cobb angle (36.01 ± 13.12°, range 20 - 58°) than those with the AG genotype (28.92 ± 7.43°, range 20 - 51°, P = 0.007), indicating that SNP rs708567 is significantly associated with the curve severity of scoliosis (Table 3).

**Table 3:** Association of the IL-17RC gene polymorphism with curve severity in AIS patients who had reached skeletal maturity

**SNP**	**Genotype**	**Number of Subjects**	**Mean Maximum Cobb Angle (Mean ± SD)**	**P-value**
rs708567	GG	215	36.01 ± 13.12	0.007
	AG	26	28.92 ± 7.43	

A value of P < 0.05 (one-way analysis of variance) was considered to be statistically significant.

## Discussion

Previous population studies have reported a significantly higher AIS incidence of 15.8% in first-degree relatives of AIS patients, 36% in dizygotic twins, and 73% in monozygotic twins [25,26]. In addition, a significantly higher incidence of AIS was found in females than males, with a female-to-male ratio of approximately 10:1[27]. Given the documented higher incidence of AIS within families and the sexual dimorphism of AIS occurrence, genetics may offer the best possibility to unravel the etiology of AIS.

A genetic association study is one of the most effective methods for identifying and characterizing the genomic variants that underlie the susceptibility to a multifactorial disease [28]. However, the quality of genetic association may be affected by many factors, such as a small sample size, population stratification, and racial and ethnic differences, and false-positive associations may be produced. For example, Zhang et al. [29] initially reported that SNP rs1256120 in the estrogen receptor β gene (ESR2) was associated with a higher susceptibility to and the curve severity of AIS in Chinese females using data from 176 AIS patients and 80 controls. However, Takahashi et al. [30] could not replicate this association in a Japanese population using data from 798 AIS patients and 637 controls. Therefore, Neale et al. [31] advocated that any positive association findings in gene-based studies should be confirmed in a different population using a larger sample size.

In a recent genetic association study, Dormans JP et al. [20] reported that SNP rs708567 (a common missense mutation) in the IL-17RC gene is associated with a higher susceptibility to AIS in a population of European ancestry, suggesting that IL-17RC may be an AIS susceptibility gene. However, in a subsequently published association study using data from 419 American AIS patients and their families, Sharma S et al. [1] did not find the association between the IL-17RC gene polymorphism and the susceptibility to AIS. Furthermore, Takahashi Y et al. [32] also did not report the association between this SNP and an AIS predisposition in Japanese female adolescents in another genome-wide association study. Therefore, it is necessary to perform an additional study to confirm the results for SNP rs708567.

In the present study, we re-evaluated the association between SNP rs705867 and the predisposition to AIS using data from a Chinese Han population. Our results show that both the GG genotype and G allele have a significantly higher frequency in AIS patients than the controls (P = 0.023 and P = 0.028, respectively). Our results are consistent with the finding of Dormans JP et al. [20], confirming that IL-17RC is a novel predisposition gene for AIS. The discrepancy between these results may reflect the issue of heterogeneity to detect modest effect sizes. In addition, our results further revealed that AIS patients with the GG genotype have a higher mean maximum Cobb angle than those with the AG genotype, indicating that the IL-17RC gene polymorphism is also associated with the curve severity in AIS patients.

The human IL-17RC gene on chromosome 3 contains 19 exons and spans 16,550 bp within the chromosomal region 3p25.3 to 3p24.1. The IL-17RC gene encodes a single-pass type I transmembrane protein (IL-17 receptor C, IL-17RC), which was characterized as an integral part of the IL-17 receptor (IL-17R) complex [21]. The IL-17R complex mediates IL-17 effector function through the IL-17 cytokine/IL-17R complex signaling axis, and current evidence indicates that both the dysfunction of IL-17RC and the IL-17 cytokine/IL-17R signaling axis are implicated in a number of human diseases. As expected, given its central role in regulating inflammation, dysfunctions in the IL-17 cytokine/IL-17R signaling axis can promote the production of several pro-inflammatory cytokines, such as tumor necrosis factor α (TNF-α), interleukin-1β (IL-1β) and interleukin-6 (IL-6), and increase the activity of metalloproteinases (MMPs), which play important roles in disc degeneration [33]. Furthermore, recent studies have also shown that an impaired IL-17 cytokine/IL-17R signaling axis drives the pathogenesis of bone destruction, in part by inducing the expression of RANKL in osteoblasts [34-36]. Thus, regarding the role of IL-17RC and/or the IL-17 cytokine/IL-17R signaling axis in disc and bone destruction, the rs708567 polymorphism may impair the function of the IL-17RC gene and accelerate disc degeneration and bone destruction, which may lead to the occurrence and progression of scoliosis. Indeed, defects within the discs and bones of AIS patients have been widely described in previous studies [37,38].

Overall, we found a positive association between SNP rs708567 and the susceptibility to and curve severity of AIS in a Chinese Han population. Unfortunately, the specific influence of SNP rs708567 on IL-17RC gene function remains unknown. As described by Dormans JP et al., SNP rs708567 is a common missense mutation (S111 L) within the IL-17RC gene, suggesting that synergistic interactions between SNP rs708567 and other polymorphisms may play an important role in impairing IL-17RC gene function. Therefore, further investigations will be performed using the method of pair-wise linkage disequilibrium (LD) analysis in our next study to clarify the possible synergistic interactions between SNP rs708567 and other polymorphisms and their effect on the susceptibility to AIS. In addition, a potential limitation that should not be ignored is the false-negative result of the Adam forward-bending test, which may lead to an ascertainment bias with regard to the controls. Therefore, further investigations using other samples are necessary to confirm this positive association.

## Conclusions

This study identified a significant association between an IL-17RC gene polymorphism and the susceptibility to and curve severity of AIS in a Chinese Han population, indicating that IL-17RC is a predisposing gene for AIS with a single thoracic curve. However, further studies are necessary to confirm these positive findings in other ethnicities.

## Competing interests

The authors declare that they have no competing interests.

## Authors’ contributions

SZ, XSQ and ZZZ performed the genetic studies. WFW recruited patients and was involved in the clinical part of the investigation. ZL performed the statistical analysis. YQ conceived the study, participated in its design and coordination and helped to draft the manuscript. All of the authors read and approved the final manuscript.

## Pre-publication history

The pre-publication history for this paper can be accessed here:

http://www.biomedcentral.com/1471-2474/13/181/prepub
